# Towards the Recognition of Interdisciplinary and Transdisciplinary Researchers

**DOI:** 10.1007/s11024-025-09578-y

**Published:** 2025-02-28

**Authors:** Mikko Salmela, Bianca Vienni-Baptista, Kirsi Cheas

**Affiliations:** 1 University of Helsinki, Helsinki, Finland; 2 ETH Zürich, Zürich, Switzerland; 3 University of Vaasa, Vaasa, Finland

**Keywords:** Interdisciplinarity, Transdisciplinarity, Problem of recognition, Academic identity, Queer, Science policy

## Abstract

Interdisciplinary and transdisciplinary research are widely considered necessary to addressing complex, often called ´wicked´, problems. Moreover, national and international funding schemes, institutional structures, and education programs have been created to foster interdisciplinary and transdisciplinary research. However, there is a largely silenced ´wicked´ problem in the heart of interdisciplinary and transdisciplinary research: the precarious situation of academics engaging in such research in their individual work. Relying on STS and ID/TD scholarships, we identify the institutional, social, cultural, and psychological challenges of interdisciplinary and transdisciplinary researchers in the contemporary scientific community. Based on Caniglia and Vogel (2023), we compare the position of these researchers to that of queer people in a heteronormative and sexually binary society. We argue that the challenges of interdisciplinary and transdisciplinary scholars, and their queer-like status, should be conceptualized as a problem of recognition of these scholars. Following Fraser (2003), we understand denial of recognition –either through maldistribution of resources, or misrecognition of identity, or both– as a set of obstacles in equal participation in academic life. Finally, we distinguish between social and institutional recognition, concluding that while researchers can contribute to social recognition through their own actions, institutional recognition requires science policy interventions by research institutions and funders.

## Introduction

Interdisciplinary and transdisciplinary (henceforth ID and TD)^1^ research are widely considered necessary to addressing a variety of complex, often also called “wicked”, problems (e.g. sustainability, global inequality, political extremism) (Hirsch Hadorn et al. 2008; Klein 2021; Felt et al. 2016). Ideals such as “co-production” (Jasanoff 2006), “Mode 2 science” (Gibbons et al. 1994), “post-normal science” (Funtowicz and Ravetz 1993) and “triple helix” (Etzkowitz and Leydesdorff 1995) urge researchers to break disciplinary boundaries in order to produce socially relevant knowledge for solving real-world problems together with other societal actors (Frodeman 2013; Klein 2021; Nowotny et al. 2001; Weingart 1997). Accordingly, national and international funding schemes, institutional structures, and education programs have been created to foster ID and TD research (Vienni-Baptista et al. 2020a).

However, there is a largely silenced wicked problem in the heart of ID and TD research itself: the precarious situation of researchers who engage in ID or TD research in their individual work, in addition to collaborating with others. These researchers are in many respects in an inferior and unprivileged position in comparison with disciplinary researchers in contemporary academia, as the former do not belong to any single disciplinary community and therefore lack an endorsed (or any) disciplinarily defined academic identity. They are confronted with pervasive challenges that are then reproduced in collective ID or TD settings (Felt et al. 2016). Authors like Julie Thompson Klein highlighted this problem in her seminal work in 1990, stating the tensions a solo researcher could face when confronted with boundary-crossing work (Klein 1990; see also Klein 2021). Among those, individual researchers require supportive institutional environments that allow them to work in multiple disciplines, accompanied by a fair evaluation system that rewards risky or unconventional interdisciplinary career paths (Felt et al. 2013; Lindvig 2018; National Academy of Sciences 2005).

To elaborate on this pervasive wicked problem, we consider interdisciplinarity and transdisciplinarity in their multiple definitions, avoiding a unique understanding of the terms that apply a restricted sense of what implies to work individually in an interdisciplinary or transdisciplinary setting.

STS and ID/TD scholarships have highlighted the mismatch between interdisciplinarity-promoting science policy and the prevailing norms of discipline-based scholarship (for instance Lyall 2019; Felt et al. 2016; Smolka et al. 2021). This mismatch translated into “a manifest misalignment between the high-level strategic pronouncements that institutions such as universities and research funders make about wanting to support interdisciplinarity and the actuality of what it means to be an interdisciplinary researcher trying to forge an academic career and scholarly identity.” (Lyall 2019: 1). Prior studies have coined the term "paradox of interdisciplinarity" to describe the scenario where interdisciplinary research is encouraged at the policy level but receives inadequate recognition in terms of funding and academic structures (Weingart 2000; Woelert and Millar 2013). Within STS, researchers have examined various aspects of this paradox. They have underscored how the disciplinary structure of academia tends to prioritize disciplinary knowledge production, researcher training, and the recruitment of faculty for tenured positions, most of which are within disciplinary degree programs (Weingart 2014; Donina et al. 2017). These disciplinary biases extend to established practices in research funding and evaluation, which typically favor disciplinary research over interdisciplinary (ID) and transdisciplinary (TD) research (Woelert and Millar 2013; Bromham et al. 2016). Scholars in STS have discussed these challenges as institutional, social, cultural, and psychological challenges faced by ID and TD researchers in contemporary academia (e.g., Parker and Crona 2012; Bridle et al. 2013; Turner et al. 2015; Felt et al. 2016; Mansilla et al. 2016; Müller and Kaltenbrunner 2019; Vienni-Baptista et al. 2020b; Smolka et al. 2021; Fini et al. 2022).

Expanding upon this existing body of research, we introduce a novel approach to understanding the paradox of interdisciplinarity — and transdisciplinarity — by centering on the perspectives of individual ID and TD researchers. We contend that previous studies have overlooked the experiences of these researchers, resulting in *hermeneutic injustice*. This injustice arises when there is a gap in collective epistemic resources of the scientific community that unfairly disadvantages certain groups or individuals in comprehending their unique and important experiences (Fricker 2007). Drawing insights from political philosophy and gender studies, we propose framing the marginalized status of ID and TD researchers within academia in normative terms as a form of *denial of recognition*, akin to the marginalization faced by queer individuals. This reframing underscores the political implications of the situation, highlighting the need for proactive measures. We advocate for action from ID and TD researchers themselves, as well as from disciplinary communities, academic institutions, and funders responsible for supporting the work and career opportunities of these researchers. As a first step for action, this article conceptualizes the constraints that individual ID and TD researchers face by raising awareness on the dimensions and dynamics of this problem.

The article aims to contribute critically to the discourse surrounding the promotion of interdisciplinarity and transdisciplinarity in science and research funding policies. We begin from examining existing research on the challenges faced by individual ID and TD researchers, including institutional, social, cultural, and psychological barriers that hinder their having an equal status with disciplinary researchers and a positive academic identity. Despite advancements in well-funded interdisciplinary fields like sustainability science, environmental sciences, cognitive science, and synthetic biology, we argue that many ID and TD researchers in the social sciences and humanities, particularly those who work alone, encounter significant difficulties. The same problems concern many team researchers as well (Pedersen 2016; Vienni-Baptista et al. 2020a; Spaapen et al. 2020). We then argue that these challenges reflect a denial of recognition as scholars with equal status and opportunities in the academic communities in which these researchers work (Honneth 1995; Fraser 2000; 2003). We show why the problem of recognition of ID and TD researchers cannot be resolved by the standard approach to distributive injustice in liberal democratic societies that relies on improving equality of opportunity for the neglected group (Sandel 2020). The problem is that the rules of the game in academia are fundamentally disciplinary and therefore discriminate against ID and TD researchers by default. Therefore, we conclude by suggesting that ID and TD researchers must assert their marginalized identities and take pride in them, akin to how queer individuals challenge heteronormative norms (Caniglia and Vogel 2023). A strong academic identity can empower ID and TD researchers in their quest for recognition. However, a positive academic identity cannot be achieved without institutional recognition by academic institutions and funding agencies, and concrete measures are needed to promote this aim (Kastenhofer and Bauer 2023).

### Institutional, Social, Cultural, and Psychological Challenges of Inter- and Transdisciplinary Researchers in Contemporary Academia

In this section, we first introduce the terms multidisciplinarity, interdisciplinarity, and transdisciplinarity and their key differences, drawing from extant literature. We then distinguish between collaborative and individual ID or TD research, and introduce the challenges that individual ID and TD researchers face in the contemporary disciplinarily structured academia, dividing these challenges into three groups: institutional, social and cultural, and psychological barriers.

Interdisciplinarity and transdisciplinarity have converted into phenomena with many roles. When facing the challenge of defining ID and TD, several authors agree that these concepts still represent contested discourses (Barry et al. 2008; Klein 2021; Lury 2018; Lyall 2019). Those attempts reveal an interwoven set of references that have different levels of understanding of what ID and TD constitute. Efforts to categorize and conceptualize the processes and outcomes of collaborative research depend fundamentally on the distinctions ranging from unidisciplinary to transdisciplinary scientific collaboration (Vienni-Baptista et al. 2020a). In a recent study, authors embraced the heterogeneity of definitions of ID and TD as a means to work productively with different understandings (Vienni-Baptista et al. 2022a). This diversity constitutes an asset rather than a drawback to conduct these research formats (Klein 2021; Vienni-Baptista al. 2022a). Against this background, the plurality of definitions may be understood as expressing the diverse aims or purposes that researchers pursue when practising inter- and transdisciplinary research, and when defining these terms (Vienni-Baptista et al. 2020a). Nevertheless, in this paper we argue that the different understandings of ID and TD and the discussion on definitions might also play a role in the lack of legitimation of individual ID and TD researchers.

As a general understanding, *interdisciplinarity* involves bodies of knowledge derived from more than one discipline in order to integrate perspectives, data or theories (Klein 2021). The following quotation asserts that

Interdisciplinarity is a means of solving problems and answering questions that cannot be satisfactorily addressed using single methods or approaches. Whether the context is a short-range instrumentality or a long-range reconceptualization of epistemology, the concept represents an important attempt to define and establish common ground (Klein 1990: 196).

For its part, *transdisciplinarity* is understood as a reflexive, integrative, method-driven scientific principle, with an emphasis on solving societal problems by integrating scientific and social bodies of knowledge (Lang et al. 2012). Transdisciplinarity aims at co-creating knowledge between researchers and other societal actors, while in some cases transgressing boundaries between disciplinary knowledge (Klein 2021).

These terms can be applied to all sorts of boundary-crossing collaboration (Klein 2021), sometimes even by funders. When the general term for all sorts of cross-disciplinary collaboration is *multidisciplinarity*, there is a threat that the requirement of integration is dropped. Instead, researchers tend to break up problem tasks into subtasks they can fulfil largely independently by following the epistemic standards and practices of their disciplines without needing to rethink and modify them (Klein 1990). Unfortunately, the multidisciplinary approach fails to account for the complexity of the ‘wicked’ problem by dividing it into neat subproblems, each of which is addressed separately. How to make researchers engage in “genuine” or “strong” integrative inter- or transdisciplinary collaborations remains a central challenge. This has been observed, for example, by LERU, in their most recent report stressing “the need for further efforts to move from programs that are multidisciplinary towards integrating knowledge from different disciplines in interdisciplinary programs and towards working with stakeholders (which is defined as transdisciplinarity).” (Wernli and Ohlmeyer 2023: 3).

Already in the 1970s, the OECD recognized, in a foundational workshop, that interdisciplinarity and transdisciplinarity can be pursued by either individual researchers, or by research teams with members from different disciplines or practices (Apostel et al. 1972). The paradigmatic and most studied form of ID and TD research is *collaborative* ID where experts from “different disciplines come together to bring their insights to a problem” (Calvert 2016: 202). Collaborative interdisciplinary work is often fraught with a variety of tensions: epistemic, structural, cultural, and emotional (Hackett 2005; Mansilla et al. 2016; Parker and Crona 2012; Rhoten and Parker 2004; Turner et al. 2015). *Epistemic tensions* involve conflicts between disciplinary standards and values, particularly regarding what constitutes reliable or meaningful research questions, methods, or results from the perspective of each discipline or domain (Andersen 2016; Mansilla et al. 2016; Turner et al. 2015; Kuhn 1970). *Cultural tensions* arise from differing worldviews and interpretations of research problems or interventions, leading to challenges in synthesising truth claims, affects, and ethical orientations into cohesive practices (Rabinow and Bennett 2009). *Structural tensions* are conflicts in interests arising from the academic reward and funding structure that generally favor disciplinary merits over interdisciplinary achievements in promotion and tenure evaluation (Müller and Kaltenbrunner 2019; Wright and Ville 2017). These tensions—epistemic, cultural, and structural—frequently trigger emotional tensions when the security of disciplinary identity clashes with the demands of interdisciplinary collaboration (Becher and Trowler 2001; Collins 1998; Mansilla et al. 2016; Parker and Hackett 2012; Salmela and Mäki 2018). Such emotional tensions may manifest as feelings of inadequacy or discomfort when venturing beyond one's disciplinary comfort zone, or as perceptions of disrespect and mistrust due to disparities in the valuation of different epistemological frameworks, often associated with financial and status discrepancies between disciplines (Callard and Fitzgerald 2016; Mansilla et al. 2016; Turner et al. 2015).

*Individual ID*, defined as "where one person integrates perspectives from different disciplines in their work" (Calvert 2016: 202, following Klein 1990), has the advantage of circumventing the interpersonal tensions inherent in collaborative ID and TD endeavours, as researchers educated in ID and TD programs excel in synthesizing diverse bodies of knowledge (Lyall 2019). While these researchers may encounter occasional challenges in navigating different epistemic standards, cultural meanings, or structurally relevant choices, these issues do not typically lead to the interpersonal tensions characteristic of collaborative ID or TD. However, individual ID and TD researchers face other significant challenges in contemporary academia.

First, *institutional* challenges faced by ID and TD researchers include the challenge of acquiring expertise in multiple disciplines and developing skills to engage with stakeholders or policymakers. These are substantial investments of time and effort (Rogga and Zscheiscler 2021; Guimarães et al. 2019; Killion et al. 2018; Enengel et al. 2012). Moreover, individual ID and TD researchers encounter challenges in forming collaborations and finding mentors across disciplines (e.g., Dooling et al. 2017; Pfirman and Martin 2017). Despite these obstacles, ID and TD researchers are motivated to make these additional efforts because the problems they tackle often transcend disciplinary boundaries or the scientific community.

Vienni-Baptista et al. (2022b) propose a practical framework based on the challenges and main questions to be considered when institutionalizing ID and TD. The framework is based on fifteen case studies from Asia, Africa, Australia, Europe, Latin America, and North America that described how ID and TD have been institutionalized in higher education. It was built with the aim to highlight the constrains individual researchers and teams face when they work in inter- or transdisciplinary settings. The dimensions of the framework take into consideration the cultural linkages and intersections between ID and TD as broad systems of meanings that structure and are structured by discourse and practices. The organizational dimension shows the structural context in which institutionalizing processes take place together with communities of practice and the strategic insights and successful aspects that can be implemented to promote ID and TD in future scenarios. The organizational dimension contributing to institutional recognition of ID and TD researchers further involves (i) identifying the features of the structural context in which the institutionalizing process takes/took place; (ii) communities of practice involved or to be consolidated; (iii) practices and policies influencing the institutionalization process; (iv) influence of impact and excellence; (v) funding schemes supporting or hindering the institutionalizing process; and (vi) types of institutional arrangements. The framework assesses the factors that act as positive or negative on institutionalizing processes depending on the context. It seeks to function as a useful tool in moving forward towards a more inclusive academia for ID and TD scholars by acknowledging the many challenges that need to be addressed at the institutional level.

Ideally, *ID or TD capital* (comprising communicative, methodological, theoretical, and collaborative skills) is recognized as valuable for addressing complex issues. Kalalahti and Muhonen (2023) who introduced this concept suggest that possessing ID and TD capital potentially paves the way for a distinguished and successful research career. However, striving for such capital comes with the risk of falling outside the traditional academic career path in which a research career may become a series of research-specific, fixed-term projects, as also noted by Kalalahti and Muhonen (2023). Moreover, disciplinary communities often fail to appreciate the efforts invested in building ID or TD capital, dismissing ID and TD research as superficial, “the dabbling of a dilettante rather than the work of a serious scholar” (Lattuca 2002: 720), or "disciplinary tourism" (Mills 2010: 71), or expressing scepticism about the ability of ID to offer credible alternatives to disciplinary frameworks in academia (Jacobs 2014).

These sceptical comments stem from fundamental misunderstandings regarding the nature of ID and TD research and the individuals engaged in these pursuits. The successful integration of knowledge from various disciplines or the collaborative creation of knowledge with societal partners does not necessarily require the formal institutional structures found in established disciplines with dedicated journals, conferences, and associations—although such structures have emerged in certain fields, such as sustainability science, cognitive science, political psychology, and synthetic biology. Moreover, ID and TD researchers specialize in “detailed understanding of how to make different forms of knowledge work together synergistically, not simply academic ´generalists´” (Lyall 2019: 66). Nevertheless, the canonical narrative of the single discipline expert prevails as Cuevas-Garcia (2016) observes.

Furthermore, academic roles and recognition systems are predominantly structured around disciplines, posing challenges for the careers of ID and TD researchers (Bridle et al. 2013; Fini et al. 2022; Salmela et al. 2021). The majority of permanent academic positions, especially in the social sciences and humanities, focus on teaching and require a track record of disciplinary teaching experience and publications in distinguished disciplinary journals. ID and TD scholars often find themselves on lengthy research-only contracts due to their lack of teaching credentials. However, without such teaching experience, they face difficulty securing disciplinary positions, which constitute the majority of tenured positions, particularly in the social sciences and humanities (Wehrli and Ohlmeyer 2023). Moreover, publications by ID and TD researchers seldom appear in top disciplinary journals as these typically do not publish interdisciplinary work (Lyall 2019). Those working in ID or TD departments or newly established centers also encounter the challenge of temporary contracts, as these positions are rarely tenured, despite the recognition of their valuable ID or TD contributions by the institution. Additionally, such organizational structures tend to be institutionally fragile and temporary, intensifying the struggle to navigate periodic evaluations and limited resources (Vienni-Baptista and Klein 2022).

Worse still, highly accomplished ID researchers are actively punished by disciplinary evaluation panels for tenured academic positions as they are viewed as threats to the disciplinary identity and knowledge domain. These issues represent *social and cultural obstacles.* Fini et al. (2022) discovered that this phenomenon was more pronounced in smaller, distinct disciplines such as typical humanities disciplines, particularly when the evaluators were representative of the focal discipline. Consequently, ID and TD researchers are rarely able to win their disciplinary peers in competition for tenured academic jobs. As a result, they are dependent on highly competitive research funding, provided they can attain it. However, many review panels are discipline-specific, and even in multidisciplinary panels, there is a tendency to prioritize the autonomy of each discipline, often resulting in bias against ID projects and applicants (Lamont 2009; Bromham et al. 2016). Although funding for ID and TD research has increased in recent years, there are still challenges in evaluating such applications. Evaluation of ID and TD research requires more effort, training and new uniform evaluation criteria compared to the traditional discipline-specific evaluation (Wernli and Ohlmeyer 2023: 29). Specific evaluation criteria have been suggested by Klein (2008) and, more recently, by Pohl et al. (2021) who proposed a more nuanced understanding of the concept of integration that includes processes at the affective and social levels besides a cognitive dimension. Still, further work on evaluation criteria that recognize excellence in ID and TD is needed to address the tensions mentioned above. While the evaluation of ID and TD career paths is still a hot topic for some funding agencies and universities, some countries such as The Netherlands and Switzerland are applying the DORA principles and implementing narrative CVs. These are promising pathways as researchers can decide on which qualitative achievements to build their profiles (Laursen et al. 2022). Even so, in some cases, ID and TD researchers find themselves in situations where they must give their research ideas and plans to their tenured colleagues who receive credit for those projects, while they themselves have to accept fixed-term positions within the project. The toll of this precarious, inferior, and humiliating low-status position of ID and TD researchers is their lack of a positive academic identity.

The traditional concept of an academic as socialized into the values, norms, practices, and belief systems of their specific epistemic community and disciplinary culture (Becher and Trowler 2001) has faced challenges due to shifts that have undermined the structural and cultural foundations of academic identities. These changes stem from shifts in higher education policy, funding, and governance, including massification, universalism, neoliberalism, new public management, and globalization (Henkel 2010; Kastenhofer and Bauer 2023; Ylijoki and Ursin 2013). However, despite these transformations, the discipline remains the primary unit of higher education, playing a significant role in shaping academic identity (Henkel 2010). While disciplinary education fosters a robust, positive academic identity grounded in disciplinary language, norms, skills, competencies, attitudes, and self-perception (Parry 2007; Schoenberger 2001), education or career development in ID or TD fields often leads to an identity crisis—a fundamental *psychological* challenge for ID and TD researchers. They often express feelings of losing their disciplinary identity, reduced confidence in their scholarly identity, the risk of being perceived as "amateurs" by disciplinary peers, insecurity, identity fatigue, flexibility in assuming multiple and shifting identities, or being a “perpetual fence-sitter” between disciplines. Consequently, becoming inter- or transdisciplinary does not result in an empowering, positive identity of an ID or TD academic. This is because such identities are undervalued and seen as anomalous compared to the prestigious disciplinary identities that continue to define the paradigm of academic expertise (Lyall 2019; Cuevas-Garcia 2016).

## Inter- and Transdisciplinary Researchers as Queers of the Academic World

In this section, we build an analogy between ID and TD researchers and queers, highlighting how the various challenges of these researchers identified in the previous section place them into positions that are in many ways similar to those of queers.

Guido Caniglia and Coleen Vogel (2023) draw parallels between TD sustainability researchers and queer individuals in a predominantly heteronormative and gender-binary society. These researchers combine their diverse expertise from various disciplines and/or social practices, often in unique ways shaped by their individual research interests and profiles, consistently challenging the disciplinary boundaries of academia. In a similar vein to queer individuals who defy social norms and advocate alternative scripts to flourish in a world hostile to those who lie outside what is pre-defined as ‘normal’ and ‘appropriate’, TD researchers navigate a world that often marginalizes those outside perceived norms. Caniglia and Vogel argue that both queer individuals and TD scholars share experiences of disorientation and embrace the transgression of established boundaries. They propose that "queer theory can help transdisciplinary sustainability researchers to raise questions that intensify the transgressive orientations of their work when contributing to just and equitable sustainability transformations" (Caniglia and Vogel 2023: 167). Furthermore, they extend this idea to all TD researchers, suggesting that "queering TD sustainability research implies a commitment to including, giving voice, and empowering queer and marginalized actors in TD spaces and institutions" (ibid.: 171).

We agree with Caniglia and Vogel´s conclusion. However, we contend that the comparison between queer individuals and ID and TD researchers runs deeper than initially perceived. The marginalized status of queer individuals in a heteronormative and binary gendered society, which they boldly challenge, bears resemblance to the situation of many ID and TD researchers within a disciplinarily structured academic community. These researchers creatively and bravely transgress its norms and boundaries, yet without acknowledgment of their existence and rights (Vienni-Baptista et al. 2020a). Importantly, academic identities are institutionalized in the disciplinary structure of academia, which makes their negotiation as hazardous for ID and TD researchers as it is for queers to navigate gender identities. Moreover, queer and ID/TD researchers face parallel challenges in contemporary academia, including difficulties in publishing in journals that do not include their scholarship and feeling marginalized in faculty ranks and epistemically due to their research being underfunded and undervalued (Winters and Ningard 2023). Whereas queer gender identity – insofar it is possible to talk about an identity that is non-essential, provisional, and fragmented (Sullivan 2003: 50) – is based on subjective identification, which is also the sufficient condition for it, ID and TD academic identities – that similarly to queer identity, are often more fluid and negotiated (Cuevas-Garcia 2016: 313) – require training or career development that equips the researcher for ID or TD research, the realization of which is an objective condition for the adoption of an ID or TD academic identity. Despite these differences, the similarities in subordination and the transgression of conventional norms and boundaries between these groups remain significant (Table 1).

**Table 1:** Similarities and differences between inter- and transdisciplinary researchers and queers

Similarities	Differences
Marginalized position and low status	Scope of marginalized position and low status: global for queers vs. local for inter- and transdisciplinary researchers
Identity basis: experiences of disorientation and transgression of conventional norms and boundaries	Identity basis: subjective identification (queers) vs. training or career development (ID and TD researchers)
Identity negotiation: hazardous	Scope of hazardous identity negotiation: personal identity for queers vs. professional identity for inter- and transdisciplinary researchers
Actionable strategies: creativity and courage, alternative scripts of flourishing, peer support	Actionable strategies: individual and joint demonstrations of gender identity in claiming recognition; practices of peer support in several domains of life (queers) vs. individual efforts with some forms of peer support to navigate the institutional struggles and lack of recognition (inter- and transdisciplinary researchers in the current situation)

Queer activism has long challenged and disrupted the oppressive structures and norms that perpetuate the marginalization and discrimination of queer individuals in today's societies (Sullivan 2003). Likewise, ID and TD researchers need to mobilise to liberate themselves from their status as the queers of the academic world. The first step towards emancipation involves an accurate conceptualization and articulation of the plight of ID and TD researchers, focusing on the problem of recognition.

## The Problem of Recognition of Interdisciplinary and Transdisciplinary Researchers

This section introduces our original conceptualisation of the challenges of ID and TD researchers in the disciplinary structured academia as a problem of recognition. Following Nancy Fraser, we distinguish two dimensions in recognition, distribution of resources or recognition of identity, and introduce participatory parity, equal opportunity to participate on a par with others in social life, as her leading ideal of social justice. We then show how ID and TD researchers are unable to participate in academic life on a par with their disciplinary peers. Instead, they suffer from injustice in terms of both recognition of identity and distribution of resources. Finally, we distinguish between two forms of recognition: social recognition, which focuses on the intersubjective recognition of identities, and institutional recognition, which influences researchers' identities through the distribution of resources and opportunities. We argue that while ID and TD researchers can contribute to their social recognition through practices of mutual support and knowledge sharing, the institutional recognition of ID and TD scholars requires active measures from academic institutions and research funding agencies.

We contend that the institutional, social, cultural, and psychological challenges encountered by ID and TD researchers in their academic careers represent an injustice against these scholars, as equal status and opportunities, as well as a positive academic identity are fundamental rights for all researchers. We identify this injustice as the denial of recognition for ID and TD researchers and their academic identities. This issue encompasses both forms of recognition outlined by Taylor (1994): the recognition of *universal dignity* and the recognition of *difference*. The recognition of universal dignity entails granting equal rights and opportunities to all individuals, regardless of their gender, ethnicity, race, or religion. Conversely, the recognition of difference entails respecting the unique characteristics of various societal groups, particularly marginalized groups such as women, people of color, and sexual minorities, who have historically been prevented from cultivating a positive and empowering social identity due to systemic oppression (Emcke 2000; Appiah 2005).

If we apply the distinction between recognition of universal dignity and recognition of difference to the academic context, we can understand the former as a requirement to grant equal rights and opportunities for pursuing a successful career to all academics, irrespective of their disciplinary, ID, or TD orientation, whereas the latter can be understood as demand of equal respect for the distinctive features of disciplinary, ID, and TD research, researchers, and their academic identities. Examples include availability of tenure track positions, and reasonably flexible access criteria to such positions, for different kinds of researchers – also recognizing possible differences between ID and TD researchers in these respects. Using the gender analogy, similarly to cis-gendered people who should embrace others with different gender identities, disciplinary researchers should embrace their ID/TD students and colleagues. This is a challenge if disciplines are tribes that protect their territories, as Becher and Trowler (2001) conceptualized. But it is nevertheless a meaningful aim.

Following Fraser (2000, 2003), we understand recognition as a matter of justice that relates to social status, not as an issue of self-realisation like Honneth (1995) and Taylor (1994) in their theories of recognition. This means examining whether some individuals or groups are being denied the status of full partners in social interaction as a consequence of institutionalized patterns of cultural value. In the status model of recognition, injustice can take two forms: maldistribution of resources or misrecognition of identity, or – typically – some combination of them. In both cases, what is at stake are obstacles in equal opportunity to participate on a par with others in social life. In maldistribution, some existing economic arrangements deny a group of people the necessary objective, i.e. material conditions for participatory parity, whereas in misrecognition, institutionalized patterns of cultural value deny them the necessary intersubjective conditions of equal participation. The intersubjective condition of recognition requires upward revaluation of misrecognized identities and deconstruction of the value patterns on which the misrecognition is based, whereas the objective condition of recognition requires removal of economic barriers of participation through redistribution of resources. Participatory parity is also the criterion for distinguishing warranted from unwarranted claims of recognition or redistribution according to Fraser. If the current arrangements prevent the claimants from participating on a par with others in social life, their claims are warranted.

Applying Fraser’s analysis to academic context, it is clear that ID and TD researchers are unable to participate in academic life on a par with their disciplinary peers. Instead, they suffer from injustice in terms of both recognition of identity and distribution of resources. They are being denied equal access to vital resources – tenured positions and research funding – for pursuing their academic careers compared to their disciplinary peers, and their academic identities are devalued in the institutionalized value structure of academia that valorizes disciplinary expertise as the only academic expertise and disciplinary identities as the only prestigious academic identities. Indeed, there is an inconsistency in the institutionalized value structure of academia, for the collaborative ID research of disciplinary experts is appreciated, whereas individual ID and TD research are being depreciated and disdained.

To explain this inconsistency, the epistemic and societal virtues of ID and TD research are the same for both collaborative and individual forms of such research. These virtues include conceptual and methodological integration, cross-fertilization, and practice relevance. Additionally, the skills and competences needed for various roles, like knowledge broker, change agent, and integrator, are crucial in both collaborative and individual ID and TD (Hoffmann et al. 2022). Therefore, it is inconsistent if these virtues and competences are valued in collaborative ID and TD, but not in individual ID and TD. This inconsistency is even more apparent considering that the required virtues and competences are often more challenging to acquire in the individual case. It is also an injustice in terms of distribution of resources against these researchers that they are not offered equal career opportunities in comparison with their disciplinary peers in academia. Their treatment suggests as if they were an unintended byproduct or “ill weed” of ID and TD promoting science policy. Besides pursuing ID and TD in their individual work, these researchers can bring their know-how to collaborative projects or programs. Together with the competences mentioned before, this know-how constitutes an asset for working in different collaborative settings, while it might be consolidated into a specific corpus of expertise for ID or TD research (Bammer et al. 2020).

Importantly, there are two dissimilar forms of recognition: social recognition, which focuses on the intersubjective recognition of identities, and institutional recognition, which influences researchers' identities through the distribution of resources and opportunities. ID and TD researchers can enhance their social recognition through various actions, such as mutual support and knowledge sharing. This social aspect is fostered through activities, such as (i) the consolidation of international networks representing the ID or TD communities^2^; (ii) the annual or biannual organization of international or national conferences on the topic of inter- or transdisciplinary research and teaching; (iii) developing master or PhD programs centered on ID and TD research with stakeholder collaboration. Encounters of researchers at a similar career stage allow them to share their common experiences and grievances, while senior researchers can mentor younger colleagues, aiding in their skill development and confidence building as ID or TD researchers. These forms of knowledge exchange and mutual support resemble what queer researchers term "queer sharing": the open and sincere sharing of vulnerabilities among peers for mutual empowerment and emancipation. Traditional and modern civil rights movements of black, women, gay, lesbian, queer, and so on people demonstrate the effectiveness of such social sharing of grievances. This is manifested in creating and reinforcing a collective identity and bonds of solidarity among the participants as well as motivating their collective political action for their shared goals (Britt and Heise 2000; Gould 2009; Salmela and von Scheve 2018).

Queer sharing operates on the principle of gift exchange (Mauss 1923/2000). It is crucial to differentiate this form of knowledge sharing from strategic dissemination of knowledge or networking solely for personal gain. Networking is often recommended for early career ID and TD researchers to foster confidence in their research and develop collaborative relationships across disciplinary boundaries (Briddle et al. 2013). Networking blends motives of personal interest with elements of gift exchange. While there may be expectations of reciprocity in gift exchange, these should remain implicit, as there's a "taboo of explicit bargaining" (Blau 2005: 112) against using gift exchange for personal advantage. While gift exchange is mutually beneficial, seeking personal benefit should not be the primary motivation. When knowledge sharing becomes transactional, it can lead to discomfort, whereas disinterested knowledge sharing associates with positive emotions such as trust, camaraderie, respect, and enjoyment (Berthoin Antal and Richebé 2009).

However, social recognition that operates through informal practices of knowledge sharing and mutual support is even at its best only a partial remedy to the problem of recognition. The full recognition of equal rights and opportunities of ID and TD researchers is possible only through *institutional recognition* by academic institutions such as universities, research institutes, and funders. Admittedly, there are certain fields, such as environmental and sustainability studies, whose members have been involved in ID and TD research for a longer time and whose institutional recognition in terms of permanent units as well as allocation of funding has proceeded in recent years. However, these advances should not prevent us from seeing that the institutional recognition of ID and TD researchers in most areas in the humanities and social sciences has received much less attention, as recent studies confirm (Pedersen 2016; Spaapen et al. 2020; Vienni-Baptista et al. 2020b). Instead, ID and TD researchers are generally evaluated in terms of disciplinary models of excellence where they come out as misfits or failures. To quote Lyall (2019: 53): “We train people to be interdisciplinary through their PhD but then restrict their career options because they do not have disciplinary expertise…. This seems entirely counterproductive, taking experts away from what they are good at” (see also Dooling et al. 2017; Donina et al. 2017). This assertion was also confirmed by Felt et al. (2016) who provided evidence on how individual PhD researchers had more difficulties when they had to carve a research profile in transdisciplinary settings. The National Academy of Sciences (2005, US) highlighted specific recommendations that individual interdisciplinary researchers need from institutions in order to fulfil their work. Although some countries, such as The Netherlands or Switzerland, are developing educational programs to overcome these difficulties, a systematic effort is needed to build suitable support for ID and TD researchers (Pohl et al. 2021; Spaapen et al. 2020).

## Concluding Remarks

The principle of equal opportunity is the preferred remedial strategy of justice employed in liberal democracies (Sandel 2020). This principle has been applied to improving the situation of oppressed and marginalized groups, such as women, people of color, sexual minorities, and indigenous people, in these societies. Unfortunately, the principle of equal opportunity in the scientific context does not help ID and TD researchers because it is inherently conservative and does not call into question the practices and rules according to which academics compete for merits. Instead, policies of equal opportunity take the existing practices both in liberal capitalist societies as well as in academia as given. In academia, the existing practices are disciplinary, and it is impossible for ID and TD researchers to succeed in competition for meritorious positions in those practices if they are seen as misfits or deviants who are trying to change or bend the established criteria of merit. Even though the principle of equal opportunity is widely endorsed in improving the situation of oppressed or marginalised groups, there is little awareness of this kind of exclusion of ID and TD researchers in contemporary academia. Indeed, recent evidence from Finnish universities shows that many deans embrace the rhetoric of equal opportunities as justification for ‘the new meritocracy’ emerging through the neoliberal ideal of free competition, although it is obvious that not all researchers compete for merits on an equal footing (Hokka 2023). Therefore, simply advocating for equal opportunity is not sufficient to address the challenges faced by ID and TD researchers. Instead, meaningful reforms are needed to alter the existing norms and create a more inclusive academic environment. This requires not only changing the rules of academic competition but also fostering a cultural shift that redefines research, its funding mechanisms, and its implications (Conroy 2020) (Table 2).

**Table 2:** Dimensions of equal participation in academic life that ID and TD researchers lack

A. Distribution of resources	B. Recognition of identity
availability of tenure-track academic positions	valuation of academic identity and expertise
equal opportunity to apply for long-term research funding at all career levels	ability to publish in high-ranking academic journals
fair and balanced review of job and funding applications	appreciation of skills and competences that allow individual ID and TD researchers to display the same epistemic and/or societal virtues that are valorised in collaborative ID and TD

Therefore, we conclude by outlining potential pathways for moving forward. Lyall (2019) proposes a dual approach to enhance the situation of ID researchers: the logic of intention and the logic of commitment. The former entails implementing clear strategies to foster interdisciplinarity, while the latter involves overcoming academic and administrative obstacles to interdisciplinarity (and transdisciplinarity), including the responsibility of funders whose priorities influence career trajectories into specific directions (Graf 2019). These avenues for change are significant, and we will delve into them further shortly. However, before doing so, we introduce a third strategy: supporting a positive academic identity for ID and TD researchers. A valued identity can provide these scholars with the resilience and drive to advocate for equal recognition in academia. Parallels can be found in traditional and modern civil rights movements where identity transformation was the first step towards emancipation from oppression and marginalization (e.g., Britt and Heise 2000; Gould 2009; Salmela and von Scheve 2018). This identity work not only aids ID and TD researchers in navigating the current academia but also holds potential as a foundational aspect of institutional reform. For a positive academic identity cannot be achieved without institutional recognition by academic institutions and funding agencies, and concrete measures are needed to promote this aim.

As we have shown above, one dimension in which ID and TD researchers are disadvantaged compared to their disciplinary colleagues concerns their access to tenured teaching posts, a situation that is not easily remedied in the short term. Nor is this entirely necessary: disciplines provide the basic research that is utilized and applied in ID and TD research. But if teaching positions go mainly to researchers working in the traditional core areas of the disciplines, this situation should be compensated for ID and TD researchers in some other ways. One solution could be permanent research-focused academic posts for ID and TD scholars that would allow these researchers to focus on their distinctive ID and/or TD contribution and skills that they could also teach to others. Another solution could be a reform of allocating research funding. ID and TD researchers should have better access to research projects, as currently only those in permanent teaching posts can, as a rule, apply for research projects. If project leaders could apply for longer-term funding for themselves under more funding schemes, the system would not be so biased in favor of researchers in permanent teaching posts. Instead, funding applications should also recognize other types of excellence in research, such as high-ranking publications within a wider disciplinary or interdisciplinary range. The evaluation process for research projects should also be reformed so that small, distinctive disciplines could not favor projects in traditional core areas of disciplines to the detriment of excellent ID and TD projects. Such measures require an active involvement of funders in the formulation of application criteria and the development of application evaluation processes. Unfortunately, these measures are not likely to happen due to the interest of disciplinary researchers to protect their positions and resources. Therefore, improving the work and funding opportunities of ID and TD researchers depends heavily on funding agencies and science policy officers, who should be able to recognize that the current system still favors disciplinary scholars and excludes those researchers who embrace the call for ID and TD research most wholeheartedly. If we agree with LERU on the need to develop ways of supporting ID and TD research, we must get down to work. To move forward, we call for future research that accounts for investigating individual career trajectories in relation to the institutional, socio-cultural, and psychological dynamics that are at play in inter- and transdisciplinary research.

## Footnotes

1 We use the abbreviations ID and TD both for the nouns “interdisciplinarity” and “transdisciplinarity”, and for the adjectives “interdisciplinary” and “transdisciplinary”. The intended meaning should be clear from the context.

2 Association of Interdisciplinary Studies (AIS) in the US or the Network of Transdisciplinary Research in Switzerland, Network of Interdisciplinary and Transdisciplinary Research in Oceania, or more recently the Global Alliance for Inter- and Transdisciplinary Research (ITD Alliance) together with smaller hubs in other regions such as the Nodo de Estudios sobre Interdisciplina y Transdisciplina in Latin America pursue similar aims (Goñi Mazzitelli and Vienni-Baptista 2023)

## References

[CR1] Andersen, Hanne. 2016. Collaboration, interdisciplinarity, and the epistemology of contemporary science. *Studies in History and Philosophy of Science* 56: 1–10. 10.1016/j.shpsa.2015.10.006. 10.1016/j.shpsa.2015.10.006 27083079

[CR2] Appiah, Kwame A. 2005. *The ethics of identity*. Princeton: Princeton University Press.

[CR3] Apostel, Leo, Guy Berger, Asa Briggs, and Guy Michaud. 1972. *Interdisciplinarity: problems of teaching and research in universities*. Paris: OECD. Accessed in August 2023, https://eric.ed.gov/?id=ED061895

[CR4] Bammer, Gabriele, Michael O’Rourke, Deborah O’Connell, Linda Neuhauser, Gerald Midgley, Julie T. Klein, Nicola J. Grigg, Howard Gadlin, Ian R. Elsum, Marcel Bursztyn, Elizabeth A. Fulton, Christian Pohl, Michael Smithson, Ulli Vilsmaier, Matthias Bergmann, Jill Jaeger, Femke Merkx, Bianca Vienni-Baptista, Mark A. Burgman, Daniel H. Walker, John Young, Hilary Bradbury, Lynn Crawford, Budi Haryanto, Cha-im Pachanee, Merritt Polk, and Goerge P. Richardson. 2020. Expertise in research integration and implementation for tackling complex problems: When is it needed, where can it be found and how can it be strengthened? *Palgrave Communications*. 10.1057/s41599-019-0380-0.

[CR5] Barry, Andrew, Georgina Born, and Gisa Weszkalnys. 2008. Logics of interdisciplinarity. *Economy and Society* 37(1): 20–49. 10.1080/03085140701760841.

[CR6] Becher, Tony, and Paul Trowler. 2001. *Academic tribes and territories. Intellectual enquiry and the cultures of discipline*. Second Edition, Society for Research into Higher Education and Open University Press imprint.

[CR7] Berthoin Antal, Ariane, and Nathalie Richebé. 2009. A passion for giving, a passion for sharing. Understanding knowledge sharing as gift exchange in academia. *Journal of Management Inquiry* 18: 78–95.

[CR8] Bridle, Helen, Anton Vrieling, Monica Cardillo, Yoseph Araya, and Leonith Hinojosa. 2013. Preparing for an interdisciplinary future: A perspective from early-career researchers. *Futures* 53: 22–32. 10.1016/j.futures.2013.09.003.

[CR9] Bromham, Lindell, Russell Dinnage, and Xia Hua. 2016. Interdisciplinary research has consistently lower funding success. *Nature* 534: 684–687. 10.1038/nature18315. 27357795 10.1038/nature18315

[CR10] Callard, Felicity, and Des Fitzgerald. 2016. *Rethinking interdisciplinarity across the social sciences and neurosciences*. Basingstoke: Palgrave Macmillan. 10.1057/9781137407962. 26726692

[CR11] Calvert, Jane. 2016. Systems biology, interdisciplinarity, and disciplinary identity. In *Collaboration in the new life sciences*, eds. John N. Parker, Niki Vermeulen, and Bart Penders, 201–218. Farnham: Ashgate.

[CR12] Caniglia, Guido, and Coleen Vogel. 2023. On being oriented: strengthening transgressive orientations in transdisciplinary sustainability research through queer theory. *GAIA* 32(1): 167–171.

[CR13] Conroy, Gemma. 2020. The push for interdisciplinary teams can lead to fake collaborations. Funding methods encourage dubious behaviors. *Nature Index*, retrieved from: https://www.natureindex.com/news-blog/push-interdisciplinary-teams-science-research-can-lead-fake-collaborations

[CR14] Cuevas-Garcia, Carlos A. 2016. *Sense-making and self-making in interdisciplinarity: an analysis of dilemmatic discourses of expertise*. PhD Thesis, Technische Universität München. https://eprints.nottingham.ac.uk/33552/1/PhD%20thesis%20final%202016.pdf

[CR15] Darbellay, Frédéric, Zoe Moody, and Todd Lubart. 2017. Creativity, design thinking, and interdisciplinarity. Springer. 10.1007/978-981-10-7524-7.

[CR16] Donina, Davide, Marco Seeber, and Stefano Paleari. 2017. Inconsistencies in the governance of interdisciplinarity: The case of the Italian higher education system. *Science and Public Policy* 44(6): 865–875. 10.1093/scipol/scx019.

[CR17] Dooling, Sarah, Jessica K. Graybill, and Vivek Shandas. 2017. Doctoral student and early career academic perspectives on interdisciplinarity. In *The Oxford handbook of interdisciplinarity* (2nd edition), ed. Robert Frodeman, 573–585. Oxford: Oxford UP. 10.1093/oxfordhb/9780198733522.013.46

[CR18] Emcke, Carolin. 2000. Between choice and coercion: Identities, injuries, and different forms of recognition. *Constellations* 7(4): 483–495. 10.1111/1467-8675.00204.

[CR19] Enengel, Barbara, Andreas Muhan, Marianne Penker, Bernhard Freyer, Stefanie Drlik, and Florian Ritter. 2012. Co-production of knowledge in transdisciplinary doctoral theses on landscape development—an analysis of actor roles and knowledge types in different research phases. *Landscape and Urban Planning* 105(1–2): 106–117. 10.1016/j.landurbplan.2011.12.004.

[CR20] Etzkowitz, Henry, and Loet Leyedorff. 1995. The triple helix – university-industry-government relations: A laboratory for knowledge based economic development. *EASST Review* 14(1): 14–19.

[CR21] Felt, Ulrike, Judith Igelsböck, Andrea Schikowitz, and Thomas Völker. 2016. Transdisciplinary sustainability research in practice: Between imaginaries of collective experimentation and entrenched academic value orders. *Science, Technology, and Human Values* 41(4): 732–761. 10.1177/0162243915626989.

[CR22] Fini, Riccardo, Julien Jourdan, Markus Perkmann, and Laura Toschi. 2022. A new take on the categorical imperative: Gatekeeping, boundary maintenance, and evaluation penalties in science. *Organisation Science*. 10.1287/orsc.2022.1610.

[CR23] Fraser, Nancy. 2000. Rethinking recognition. *New Left Review* 3(3): 107–118.

[CR24] Fraser, Nancy. 2003. Social justice in the age of identity politics: Redistribution, recognition, and participation. In *Redistribution or recognition. A political-philosophical exchange*, eds. Nancy Fraser and Axel Honneth, 7–108. New York: Verso Books.

[CR25] Fricker, Miranda. 2007. *Epistemic injustice. Power and the ethics of knowing*. Oxford: Oxford University Press.

[CR26] Frodeman, Robert. 2013. *Sustainable knowledge. A theory of interdisciplinarity*. Basingstoke: Palgrave Macmillan.

[CR27] Funtowicz, Silvio O., and Jerome R. Ravetz. 1993. Science for the post-normal age. *Futures* 25(7): 739–755. 10.1016/0016-3287(93)90022-L.

[CR28] Gibbons, Michael, Camille Limoges, Helga Nowotny, Simon Schwartzman, Peter Scott, and Martin Trow. 1994. *The new production of knowledge. The dynamics of science and research in contemporary societies*. London: SAGE Publications.

[CR29] Goñi Mazzitelli, Maria, and Bianca Vienni-Baptista. 2023. The value of regionalism in interdisciplinarity and transdisciplinarity in Latin America. *Issues in Interdisciplinary Studies* 41(1): 41–56.

[CR30] Graf, Joél. 2019. Bringing concepts together: Interdisciplinarity, transdisciplinarity, and SSH Integration. *fteval Journal for Research and Technology Policy Evaluation* 48: 33–36.

[CR31] Guimarães, Maria H., Christian Pohl, Olivia Bina, and Marta Varanda. 2019. Who is doing inter- and transdisciplinary research, and why? An empirical study of motivations, attitudes, skills, and behaviours. *Futures* 112: 102441. 10.1016/j.futures.2019.102441.

[CR32] Hackett, Edward J. 2005. Essential tensions. Identity, control, and risk in research. *Social Studies of Science* 35(5): 787–826.

[CR33] Henkel, Mary. 2010. Introduction: Change and continuity in academic and professional Identities. In *Academic and professional identities in higher education*, eds. Celia Whitchurch and George Gordon, 3–12. New York City: Routledge.

[CR34] Hirsch Hadorn, Gertrude, Holger Hoffmann-Riem, Susette Biber-Klemm, Walter Grossenbacher-Mansuy, Dominique Joye, Christian Pohl, Urs Wiesmann, and Elisabeth Zemp, eds. 2008. *Handbook of transdisciplinary research*. Zürich: Springer.

[CR35] Hoffmann, Sabine, Lisa Deutsch, Julie T. Klein, and Michael O´Rourke. 2022. *Integrate the integrators!* A call for establishing academic careers for integration experts: Humanities and Social Sciences Communications. 10.1057/s41599-022-01138-z.

[CR36] Hokka, Johanna. 2023. Emotional distance, detachment, compassion and care: The affective milieu of academic management in the neoliberal university. *The Sociological Review* 71(6): 1322–1340. 10.1177/00380261231189050.

[CR37] Honneth, Axel. 1995. *The struggle for recognition. The moral grammar of social conflicts*. Transl. by Joel Anderson. Boston: The MIT Press.

[CR38] Jacobs, Jerry A. 2014. *In defense of disciplines. Interdisciplinarity and specialization in the research university*. Chicago: University of Chicago Press.

[CR39] Jasanoff, Sheila, ed. 2006. *States of knowledge. The co-production of science and the social order*. London & New York: Routledge.

[CR40] Kalalahti, Mira, and Reetta Muhonen. 2023. Tieteidenvälinen urakehitys tutkijakollegiumien kontekstissa [Interdisciplinary career development in the context of institutes of advanced study]. In *Rakenne, toiminta, toimijuus: Korkeakoulututkimuksen XV kansallinen symposium, 15.-16.8.2023, Jyväskylä. CHERIF 2023 Symposium Program: Structure, Action, and Agency, August 15-16, 2023*. Korkeakoulututkimuksen seura ry. https://docs.google.com/document/d/1mNFCIS3kj257birlpuCMWjfS7owVPlchB-N94wlsEr0.

[CR41] Kastenhofer, Karen, and Anja Bauer. 2023. “Are you a TA practitioner, then?” – Identity constructions in post-normal science. *Minerva* 61: 93–115. 10.1007/s11024-022-09480-x. 36789005 10.1007/s11024-022-09480-x PMC9918560

[CR42] Killion, Alexander K., Kelley Sterle, Emily N. Bondank, Jillian R. Drabik, Abhinandan Bera, Sara Alian, Kristen A. Goodrich, Marcia Hale, Rachel A. Myer, Quang Phung, Aaron M. Shew, and Anastasia W. Thayer. 2018. Preparing the next generation of sustainability scientists. *Ecology and Society* 23(4): 39. 10.5751/ES-10395-230439.

[CR43] Klein, Julie T. 1990. *Interdisciplinarity: History, theory, and practice*. Detroit: Wayne State University Press.

[CR44] Klein, Julie T. 2008. Evaluation of interdisciplinary and transdisciplinary research: A literature review. *American Journal of Preventive Medicine* 35 (2 Suppl): 116–123. 10.1016/j.amepre.2008.05.010. 10.1016/j.amepre.2008.05.010 18619391

[CR45] Klein, Julie T. 2021. *Beyond interdisciplinarity. Boundary work, communication and collaboration*. New York: Oxford University Press. 10.1093/oso/9780197571149.001.0001.

[CR46] Kuhn, Thomas S. 1970. *The structure of scientific revolutions*. Chicago, IL: The University of Chicago Press.

[CR47] Lamont, Michéle. 2009. *How professors think. Inside the curious world of academic judgment*. Cambridge (MA): Harvard University Press.

[CR48] Lattuca, Lisa R. 2002. Learning interdisciplinarity: Sociocultural perspectives on academic work. *The Journal of Higher Education* 73(6): 711–739.

[CR49] Laursen, Bethany K., Nicole Motzer, and Kelly J. Anderson. 2022. Pathways for assessing interdisciplinarity: A systematic review. *Research Evaluation* 31(3): 326–343. 10.1093/reseval/rvac013.

[CR50] Lindvig, Kathrine. 2018. The implied PhD student of interdisciplinary research projects within monodisciplinary structures. *Higher Education Research & Development* 37(6): 1171–1185. 10.1080/07294360.2018.1474343.

[CR51] Lury, Celia. 2018. Introduction: activating the present of interdisciplinary methods. In *Routledge handbook of interdisciplinary research methods*, eds. Celia Lury, Rachel Fensham, Alexandra Heller-Nicholas, Sybille Lammes, Angela Last, Mike Michael, and Emma Uprichard, 1–23. London: Routledge.

[CR52] Lyall, Catherine. 2019. *Being an interdisciplinary academic. How institutions shape university careers*. Basingstoke: Palgrave.

[CR53] MacLeod, Miles. 2018. What makes interdisciplinarity difficult? Some consequences of domain specificity in interdisciplinary practice. *Synthese* 195: 697–720. 10.1007/s11229-016-1236-4.

[CR54] Mansilla, Veronica B., Michéle Lamont, and Kyoko Sato. 2016. Shared cognitive-emotional-interactional platforms: Markers and conditions for successful interdisciplinary collaborations. *Science, Technology, & Human Values* 41(4): 571–612. 10.1177/0162243915614103.

[CR55] Mauss, Marcel. 1923/2000. *The gift. The form and reason for exchange in archaic societies*. Transl. by W.D. Halls. New York: W.W. Norton & Company.

[CR56] Mills, David. 2010. Employment patterns in and beyond one’s discipline. In *Becoming an academic: international perspectives*, eds. Lynn McAlpine and Gerlese Akerlind, 71-95. Basingstoke: Palgrave.

[CR57] Müller, Ruth, and Wolfgang Kaltenbrunner. 2019. Re-disciplining academic careers? Interdisciplinary practice and career development in a Swedish environmental sciences research center. *Minerva* 57(4): 479–499. 10.1007/s11024-019-09373-6.

[CR58] Academy, National, and of Sciences, N. A. o. E., Institute of Medicine. 2005. *Facilitating interdisciplinary research*. Washington, DC: The National Academies Press.

[CR59] Nowotny, Helga, Peter B. Scott, and Michael T. Gibbons. 2001. *Re-thinking science. Knowledge and the public in an age of uncertainty*. London: Polity Press.

[CR60] Parker, John N., and Beatrice Crona. 2012. On being all things to all people: Boundary organizations and the contemporary research university. *Social Studies of Science* 42(2): 262–289. 10.1177/0306312711435833.

[CR61] Parker, John N., and Edward J. Hackett. 2012. Hot spots and hot moments in scientific collaborations and social movements. *American Sociological Review* 77: 21–44. 10.1177/0003122411433763.

[CR62] Parry, Sharon. 2007. *Disciplines and doctorates. Higher education dynamics*, Vol. 16. Dordrecht: Springer. 10.1007/1-4020-5312-6

[CR63] Pedersen, David B. 2016. Integrating social sciences and humanities in interdisciplinary research. *Palgrave Communications* 2: 16036. 10.1057/palcomms.2016.36.

[CR64] Pfirman, Stephanie, and Paula J.S. Martin. 2017. Facilitating interdisciplinary scholars. In *The Oxford handbook of interdisciplinarity* (2nd edition), ed. Robert Frodeman, 586-600. Oxford/New York: Oxford UP. 10.1093/oxfordhb/9780198733522.013.47

[CR65] Pohl, Christian, Julie Thompson Klein, Sabine Hoffmann, Cynthia Mitchell, and Dena Fam. 2021. Conceptualising transdisciplinary integration as a multidimensional interactive process. *Environmental Science & Policy* 118: 18–26. 10.1016/j.envsci.2020.12.005.

[CR66] Rabinow, Paul, and Gaymon Bennett. 2009. Human practices: interfacing three modes of collaboration. In *The ethics of protocells. Moral and social implications of creating life in the laboratory*, eds. Mark A. Bedau and Emily C. Parke, 263-290. Cambridge, MA: The MIT Press. 10.7551/mitpress/7548.003.0006

[CR67] Rhoten, Diana, and Andrew Parker. 2004. Risks and rewards of an interdisciplinary research path. *Science* 306(5704): 2046. 15604393 10.1126/science.1103628

[CR68] Rogga, Sebastian, and Jana Zscheiscler. 2021. Opportunities, balancing acts, and challenges – doing PhDs in transdisciplinary research projects. *Environmental Science & Policy* 120: 138–144.

[CR69] Salmela, Mikko, and Christian von Scheve. 2018. Emotional dynamics of right- and left-wing political populism. *Humanity and Society* 42(4): 434–454.

[CR70] Salmela, Mikko, and Uskali Mäki. 2018. Disciplinary emotions in imperialistic interdisciplinarity. In *Scientific imperialism: exploring the boundaries of interdisciplinarity*, eds. Uskali Mäki, Adrian Walsh, and Manuela Fernandez-Pinto, 31–50. London & New York: Routledge. 10.4324/9781315163673

[CR71] Salmela, Mikko, Miles MacLeod, and Johan Munck af Rosenschöld. 2021. Internally incentivized interdisciplinarity: organisational restructuring and emerging tensions. *Minerva* 59: 355–377. 10.1007/s11024-020-09431-4.

[CR72] Sandel, Michael. 2020. *The tyranny of merit. What’s become of the common good?* New York: Farrar, Straus & Giroux.

[CR73] Smolka, Marieke, Erik Fisher, and Alexandra Hausstein. 2021. From affect to action: Choices in attending to disconcertment in interdisciplinary collaborations. *Science, Technology, & Human Values* 46(5): 1076–1103. 10.1177/0162243920974088.

[CR74] Spaapen, Jack, Bianca Vienni-Baptista, Anna Buchner, and Christian Pohl. 2020. Report on survey among interdisciplinary and transdisciplinary researchers and post-survey interviews with policy stakeholders. 10.5281/zenodo.3824727

[CR75] Sullivan, Nikki. 2003. *A critical introduction to queer theory*. New York: New York University Press.

[CR76] Taylor, Charles. 1994. The politics of recognition. In *Multiculturalism and the politics of recognition*, ed. Amy Gutmann, 25-74. Princeton: Princeton UP. 10.2307/j.ctt7snkj.6

[CR77] Turner, V. Kelly, Karina Benassaiah, Scott Warren, and David Iwaniec. 2015. Essential tensions in interdisciplinary scholarship: navigating challenges in affect, epistemologies, and structure in environment- society research centers. *Higher Education*. 10.1007/s10734-015-9859-9.

[CR78] Weingart, Peter. 1997. From ‘finalization’ to ‘mode 2’: old wine in new bottles? *Social Science Information* 36(4): 591–613. 10.1177/053901897036004002.

[CR79] Weingart, Peter. 2000. Interdisciplinarity: the paradoxical discourse. In *Practising interdisciplinarity*, ed. Peter Weingart and Nico Stehr, 25–41. Toronto: University of Toronto Press.

[CR80] Weingart, Peter. 2014. Interdisciplinarity and the new governance of universities. In *University experiments in interdisciplinarity: obstacles and opportunities*, eds. Peter Weingart and Britta Padberg, 151–174. Bielefeld: Transcript Verlag.

[CR81] Wernli, Didier, and Jane Ohlmeyer. 2023. Implementing interdisciplinarity in research-intensive universities: good practices and challenges. Advice paper No. 30, March 2023. *League of European Research Universities, LERU*. Accessed on August 2023: https://www.leru.org/files/Publications/Implementing-interdisciplinarity-in-research-intensive-universities-good-practices-and-challenges_Full-paper.pdf

[CR82] Vienni-Baptista, Bianca, Catherine Lyall, Jane Ohlmeyer, Jack Spaapen, Doireann Wallace, and Christian Pohl. 2020a. Improving pathways to interdisciplinary and transdisciplinary research for the Arts, Humanities and Social Sciences: first lessons from the SHAPE-ID project – Policy Brief. 10.5281/zenodo.3824954

[CR83] Vienni-Baptista, Bianca, Maciej Maryl, Piotr Wciślik, Isabel Fletcher, Anna Buchner, Doireann Wallace, and Christian Pohl. 2020b. Final report on understandings of interdisciplinary and transdisciplinary research and factors of success or failure. 10.3929/ethz-b-000516065

[CR84] Vienni-Baptista, Bianca, and Julie T. Klein, eds. 2022. *Institutionalizing interdisciplinarity and transdisciplinarity. Collaboration across cultures and communities*. London and New York: Routledge.

[CR85] Vienni-Baptista, Bianca, Isabel Fletcher, Catherine Lyall, and Christian Pohl. 2022a. Embracing heterogeneity: Why plural understandings strengthen interdisciplinarity and transdisciplinarity. *Science and Public Policy* 49(6): 895–877.

[CR86] Vienni-Baptista, Bianca, Julie T. Klein, and Danilo Streck. 2022b. Conclusion: A comparative framework for institutionalizing inter- and transdisciplinary research and teaching in higher education. In *Institutionalizing interdisciplinarity and transdisciplinarity. Collaboration Across Cultures and Communities*, eds. Bianca Vienni-Baptista and Julie T. Klein, 232–248. London and New York: Routledge.

[CR87] Woelert, Peter, and Victoria Millar. 2013. The ‘paradox of interdisciplinarity’ in Australian research governance. *Higher Education* 66: 755–767. 10.1007/s10734-013-9634-8.

[CR88] Wright, Claire, and Simon Ville. 2017. Visualising the interdisciplinary research field: The life cycle of economic history in Australia. *Minerva* 55(3): 321–340. 10.1007/s11024-017-9319-z.

[CR89] Ylijoki, Oili-Helena, and Jani Ursin. 2013. The construction of academic identity in the changes of Finnish higher education. *Studies in Higher Education* 38(8): 1135–1149. 10.1080/03075079.2013.833036.

